# *Xylella fastidiosa* and Drought Stress in Olive Trees: A Complex Relationship Mediated by Soluble Sugars

**DOI:** 10.3390/biology11010112

**Published:** 2022-01-11

**Authors:** Mariarosaria De Pascali, Marzia Vergine, Carmine Negro, Davide Greco, Federico Vita, Erika Sabella, Luigi De Bellis, Andrea Luvisi

**Affiliations:** 1 Department of Biological and Environmental Sciences and Technologies, University of Salento, 73100 Lecce, Italy; mariarosaria.depascali@unisalento.it (M.D.P.); carmine.negro@unisalento.it (C.N.); davide.greco@unisalento.it (D.G.); erika.sabella@unisalento.it (E.S.); luigi.debellis@unisalento.it (L.D.B.); andrea.luvisi@unisalento.it (A.L.); 2 Department of Biology, University of Bari Aldo Moro, 70121 Bari, Italy; federico.vita@uniba.it

**Keywords:** abiotic–biotic stress, combined stress, plant disease, sugar transport

## Abstract

**Simple Summary:**

Carbohydrates play important roles in tolerance to both biotic and abiotic stressors. *Xylella fastidiosa*, the causal agent of “Olive Quick Decline Syndrome”, is a quarantine pathogen that induces drought stress in the host, aggravated by eventual water shortage, which is a frequent environmental condition in Mediterranean olive groves. At present, the resistance mechanisms shown by few resistant olive cultivars (e.g., *cv* Leccino) are not completely known; therefore, the aim of this research is to understand whether sugar metabolism is involved in the cross-talk mechanisms of biotic and abiotic responses. The results show that drought stress response induces effects beneficial to resistance of *Xylella fastidiosa* in *cv* Leccino. In the current context of global climate change, this study supports the importance of investigating the complex drought–disease interaction to detect resistance traits and thus find ways to counter the threat of this pathogen in the future.

**Abstract:**

*Xylella fastidiosa* (*Xf*) subsp. *pauca* “De Donno” is the etiological agent of “Olive Quick Decline Syndrome” (OQDS) on olive trees (*Olea europaea* L.); the presence of the bacterium causes xylem vessel occlusions inducing a drought stress and the development of leaf scorch symptoms, which may be worsened by water shortage in summer. In order to evaluate how the two stress factors overlap each other, the carbohydrate content and the expression patterns of genes related to carbohydrate metabolism have been evaluated in two olive *cvs* trees (Cellina di Nardò, susceptible to *Xf*, and Leccino, resistant to *Xf*) reporting transcriptional dynamics elicited by *Xf* infection, drought, or combined stress (drought/*Xf*). In the *Xf*-susceptible Cellina di Nardò plants, *Xf* and its combination with drought significantly decrease total sugars compared to control (−27.0% and −25.7%, respectively). In contrast, the *Xf*-resistant Leccino plants show a more limited reduction in sugar content in *Xf*-positive conditions (−20.1%) and combined stresses (−11.1%). Furthermore, while the amount of glucose decreases significantly in stressed Cellina di Nardò plants (≈18%), an increase was observed in Leccino plants under drought/*Xf* combined stresses (+11.2%). An opposite behavior among *cvs* was also observed for sucrose, as an accumulation of the disaccharide was recorded in stressed Leccino plants (≈37%). The different response to combined stress by *Xf-*resistant plants was confirmed considering genes coding for the sucrose or monosaccharide transporter (*OeSUT1*, *OeMST2*), the cell wall or vacuolar invertase (*OeINV-CW*, *OeINV-V*), the granule-bound starch synthase I (*OeGBSSI*) and sucrose synthase *(OeSUSY*), with a higher expression than at least one single stress (e.g., ≈1-fold higher or more than *Xf* for *OeMST2*, *OeINV-CW*, *OeINV-V*, *OeGBSSI*). It is probable that the pathways involved in drought stress response induce positive effects useful for pathogen resistance in *cv* Leccino, confirming the importance of investigating the mechanisms of cross-talk of biotic and abiotic responses.

## 1. Introduction

In nature, plants are exposed to a wide array of stress factors during their lifecycles, such as pathogens, drought, salt, cold, and heat, which limit growth and productivity. Thus, plants have developed physiological, biochemical, and molecular mechanisms to respond quickly and efficiently to stresses and acquire new equilibrium between growth and defense. Generally, these responses occur through a cascade of events that start with the perception of environmental changes and end with the expression of a broad spectrum of genes induced by the plant’s information. Carbohydrates play an essential role during plant growth and development because they are structural and storage substances, respiratory substrates, and intermediate metabolites of many biochemical processes, but they are also involved in stress tolerance as a modulator of gene expression of genes, behaving as osmoprotectants or signaling molecules in both abiotic and biotic stress tolerance [1,2]. Furthermore, it has been proven that the accumulation of sugars is a joint event under cold stress, drought, or pathogen attack [3,4,5], whereas lower sugar levels in tissues are often recorded under reduced oxygen conditions [6]. Many studies have reported a strong correlation between soluble sugar concentration and stress tolerance [5,7,8], showing that plants have developed an efficient perception and transmission system induced by low or high sugar availability.

*Xylella fastidiosa* (*Xf*) subsp. *pauca* ‘De Donno’ is a quarantine pathogen that has been recognized since 2013 in Southern Italy (Salento peninsula, located in the Apulia region), associated with the “Olive Quick Decline Syndrome” (OQDS) on *Olea europaea* L. trees [9], representing the first European evidence of this pathogen. *Xf* is an endophytic commensal and spreads from the site of infection to colonize the xylem. In contrast, the subsequent vessel occlusion [10] induces olive trees to drought stress conditions and symptom development, which may be worsened by abiotic stresses, particularly by water shortage. Therefore, the relationship between plant and water balance is particularly complex in contexts susceptible to *Xf* infections and water stress, such as Southern Italy, because the two stress factors seem to overlap each other, generating possible adverse synergies.

These symptoms are particularly severe on *cv* Cellina di Nardò plants, while a notable resistance was observed in the less common Leccino cultivar [11,12,13,14,15]. No cure is currently available, and according to several studies, the spread of *Xf* could cost billions of euros over the next years [16,17]. In addition, it is necessary to underline that the bacterium has spread in a territory, the Salento peninsula, characterized by frequent droughts [18]. A condition that in the past has favored the large development of olive growing, particularly suited to the soil and climatic conditions of the territory—as well as in many Mediterranean areas—and which has promoted a wide use of *cv* Cellina di Nardò, considered particularly resistant to drought, but which, as mentioned, is unfortunately very susceptible to the bacterium.

Therefore, it is essential to understand the basis of plant response mechanisms and the functioning of signaling pathways to detect cultivar resistance traits and thus counter the threat of *Xf*. The focus of this work is to understand how drought influences plant resistance to xylem-limited pathogens and plant responses to combined abiotic/biotic stress. Greater knowledge is useful for breeding programs aimed to improve *Xf* resistance in *O. europaea* cultivars as well as in the forecast of future climate changes. For this purpose, the changes in carbohydrate content and the expression patterns of genes related to carbohydrate metabolism in two cultivars (Cellina di Nardò and Leccino) have been investigated. We hypothesize that the resistance of Leccino to *Xf* could be related to its response to water stress, which could trim defense pathways against *Xf*, probably mediated by sugars. In particular, we report on the transcriptional dynamics elicited by infection in combination with drought to understand the mechanisms of cross-talk of biotic and abiotic responses, primarily to stem the effects that limit the growth and yield of plants and encourage the development of genetic breeding programs useful for combined stress tolerance.

## 2. Materials and Methods

### 2.1. Field Conditions and Plant Material

Trials were carried out on *O. europaea* L. plants, *cvs* Cellina di Nardò and Leccino, in orchards located in Lecce (Apulia, Southern Italy). Selected plants had previously received the same agronomic practices (with differences only in water management; see following paragraphs) and insect control over five years according to EU Decision 2015/789.

We used an experimental design with 24 olive trees, 12 *cv* Cellina di Nardò and 12 *cv* Leccino with ages ranging from 25 to 35 years. The trials were carried out on sandy soils (average soil texture parameters: 76.0% sand, 19.1% silt, 4.9% clay, and 1.9% organic matter), considering four experimental conditions: *Xf*-positive trees naturally infected and irrigated (named “*Xf*”, three plants/*cv*), *Xf*-negative trees and subjected to water deficit (named “Drought”, three plants/*cv*), *Xf*-positive trees subjected to water deficit (named “Combined”, three plants/*cv*), and *Xf*-negative trees and irrigated (named “Ctrl”, 3 plants/*cv*).

Samples were collected in the summer after four weeks of no rainfall. For the irrigated plants, the water management predicted scheduled irrigation using the water budget approach, according to Marra et al. [19]. In the month before the sampling, 300 L/tree of water were dispensed to the irrigated plots.

*Xf* infection symptoms were observed for each plant during the 12 months before sampling. The presence of symptoms was scored using a severity scale of 1 to 3 as described by Luvisi et al. [13].

The *Xf*-positive or *Xf*-negative plants were assessed by real-time PCR (qPCR) [20] in two successive years (2019–2020). To confirm the *Xf* presence in the xylem vessels of the infected plants, a fluorescence in situ hybridization-confocal laser scanning microscopy (FISH-CLSM) analysis was carried out. Several petioles (~1.5 × 1.5 cm) were excised from leaves of the infected and control plants. After the surface sterilization for 1 min in 70% ethanol, they were rinsed three times in sterile distilled water. The cuttings were fixed in 4% paraformaldehyde in phosphate-buffered saline (1× PBS) overnight at room temperature, followed by washing in PBS buffer for 10 min at room temperature. After fixation, samples were dehydrated by two successive 1-h incubations in each of 70, 80, 95, and 100% ethanol, then embedded in paraffin and cut into 45 µm-thick sections with a microtome Leica RM 2155 (Leica Microsystems, Mannheim, Germany). Sections were transferred to 1:1 (*v*/*v*) PBS:96% ethanol and maintained at −20 °C until FISH staining. To dissolve the paraffin, sections were embedded in toluene for 3 min at 43 °C. After removing the toluene, the in-tube FISH staining was performed according to Cardinale et al. [10].

Analyses reported here (RWC, carbohydrate determination, genes expression) were carried out on plant material collected from *Xf*-tested twigs. In addition, the plants selected were monitored for symptoms caused by natural infection of other pathogens, as reported by De Pascali et al. [21], excluding significant co-infections from the trial.

### 2.2. Relative Water Content Measurement

Relative water content (RWC) was carried out following the procedure proposed by Barrs and Weatherley [22] on fully expanded leaves of similar age, divided into blocks of 10 leaves per treatment. First, leaves were excised, weighed fresh (FW), and placed in distilled water in the dark for 24 h to rehydrate. Next, the turgid leaf weight (TW) was measured, then leaves were dried at 80 °C for 48 h, and dry weight (DW) was determined. The RWC was calculated as:

RWC = [(FW − DW) / (TW − DW)] × 100

### 2.3. Carbohydrate Determination

As *X. fastidiosa* is confined to the xylem tissue, the petiole from leaf samples are the best source for analysis and diagnosis as they contain a high number of xylem vessels [23]. In this work, the total soluble sugars were extracted from 500 mg of lyophilized dry petioles powder using 5 mL 80% ethanol. After 10 min of incubation at 95 °C, the extract was centrifuged at 2500 rpm for 5 min. The supernatant was assayed for soluble sugars using the phenol–sulphuric acid method where diluted extracts were shaken with 1 mL 2% phenol and 5 mL 98% sulphuric acid [24]. Once the extract had cooled, its absorbance was determined at 490 nm. Glucose, fructose, and sucrose contents were determined with a K-SUFRG kit (Megazyme) according to the manufacturer’s procedure. All measurements were performed in triplicate. Absorbance measurements were determined using UV Jasco V-550 UV/VIS Ubest spectrophotometer and the data were expressed as mg/g dry weight.

### 2.4. Total RNA Isolation, cDNA Synthesis and RT-qPCR Analysis

Total RNA was extracted from leaf samples using TRIzol^®^ (Promega, Madison, WI, USA) according to the manufacturer’s protocol. RNA samples were treated with DNase I (Promega) before their absorbance was read at 260 and 280 nm to define RNA concentration and purity. cDNA synthesis was performed using TaqMan^®^ Reverse Transcription Reagents (Applied Biosystems, Waltham, MA, USA) according to the manufacturer’s instruction, with oligo (dT)18 as a primer. The RT-qPCR was carried out using SYBR Green fluorescent detection in a real-time PCR thermal cycler (QuantStudio™ 3 Real-Time PCR System, Applied Biosystems). The PCR program was: 2 min at 50 °C and 10 min at 95 °C, followed by 45 cycles of 95 °C for 15 s and 60 °C for 1 min.

Melting curve analysis was performed after PCR to evaluate the presence of non-specific PCR products and primer dimers. Three biological and three technical replicates were analyzed. The primers used in this work have been taken from the literature (Table 1). In particular, primers designed on the genes involved in metabolism and transport sucrose were chosen: the sucrose synthase (SUSY), which plays a key role in sugar metabolism, primarily in sink tissues [25]; the cell wall invertase (INV-CW) and vacuolar invertase (INV-V), which irreversibly cleave sucrose into glucose and fructose [26]; the granule-bound starch synthase I (GBSSI), which is involved in starch biosynthesis [27]; finally, the sucrose transporter (SUT1) and monosaccharide transporter (MST2), which are involved in sugar influx [28]. In summary, these enzymes are involved at different levels in sugar loading and unloading in long-distance transport (source to sink) besides modulating stress response.

The relative abundance of the β-actin gene was used as the internal standard (Table 1). Relative gene expression levels were calculated with the log_2_ 2^−∆∆Ct^ method [29]. The efficiency of the target amplification was evaluated for each primer pair, and the corresponding value was used to calculate the fold changes (FC).

**Table 1:** List of primers used for real-time RT-qPCR.

Name	Sequence 5′-3′	Reference	GeneBank
***Oeβ-Act* F**	ACTATGAACAGGATCTTGAG	Rossi et al., 2016 [30]	AF545569.1
***Oeβ-Act* R**	GAACCACCACTGAGGACGAT		
***OeSUT1*F**	TCGGTTATGCGGCTGGAT	Sabella et al., 2019 [31]	JN656245.1
***OeSUT1*R**	CAGGCTTTTGTTTTGGTAAATGG		
***OeMST2*F**	GCCAATGTGGACGAGGAGTT	Sabella et al.,2019 [31]	DQ087177.2
***OeMST2*R**	TGCTCCACCTTCCTCGACTCT		
***OeSUSY* F**	GCCTGGACTCTACCGAGTTGTT	Alagna et al., 2016 [32]	unigene02089
***OeSUSY* R**	CACGCATAGGTGTTCCTTGTTC		
***OeINV-CW* F**	AGACAAGGCAGAGACATTCGAC	Alagna et al., 2016 [32]	unigene02494
***OeINV-CW* R**	ATGCATCAGAGCACATGAGAAC		
***OeINV-V* F**	CCAGTCAGCGAAGTGGAAGAAT	Alagna et al., 2016 [32]	unigene01665
***OeINV-V* R**	TGTAACCAGCATCAGCATCAGC		
***OeGBSSI* F**	TGTGCCAAAGTCGACCCTGCCG	Alagna et al., 2016 [32]	unigene00185
***OeGBSSI* R**	TGGTTCACTGCTGGCAGCCCC		

### 2.5. Statistical Analysis

All data were reported as the mean ± SD with at least three replications for each leaf olive sample. A two-way ANOVA with the replicates of each measure was carried out on the determination of total sugar content, followed by Tukey-HSD (honestly significant difference) post hoc test (*p* < 0.05). In addition, a two-way ANOVA with the replicates of each measure was carried out on gene expression level data using cultivar and stress conditions as main factors. The data relating to the content of glucose, fructose, and sucrose, and gene expression level for each stress (individual and combined stresses) were subjected to multiple t-test analyses (FDR = 5%). Statistical analyses were performed using GraphPad software, version 8.0.2.

## 3. Results

### 3.1. Evaluation of Xylella fastidiosa Infection and Plant Water Content

**Figure 1:**
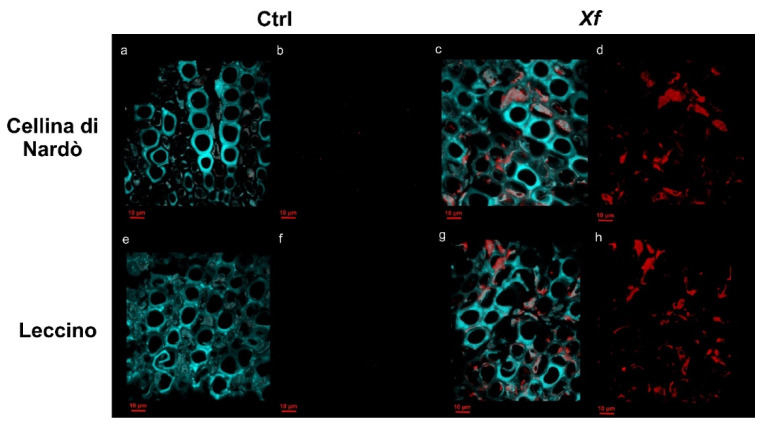
Confocal microscopy images showing the *Xylella fastidiosa* (*Xf*) colonization of xylem vessels by fluorescence in situ hybridization (FISH). Cyan: plant tissue autofluorescence; red: signal of the *Xf*-specific Cy5-labeled KO210 FISH probe; (**a**,**c**,**e**,**g**): overlap of cyan and red signals in Cellina di Nardò and Leccino control (Ctrl), and *Xf*-infected plants (*Xf*); (**b**,**d**,**f**,**h**): exclusively the red signal of the *Xf*-specific FISH probe.

The disease severity was assessed for *Xf* or combined stress plants to select homogenous trees (Cellina di Nardò 1.8 ± 0.3 and Leccino 0.7 ± 0.2). Regarding infected plants, the Cellina di Nardò and Leccino trees showed CFU/mL ranges of 2.86 × 10^6^–3.41 × 10^5^ and 1.68 × 10^5^–4.06 × 10^4^, respectively. All the analyzed petioles from infected leaves showed the xylem vessel colonization by *Xf* while no infected or occluded vessels were observed in petiole sections of Ctrl plants (Figure 1).

The RWCs of Ctrl plants were not significantly different among the analyzed cultivars (RWC ~97% for both). As expected, under the absence of irrigation, the Leccino plant showed an RWC value of about 22% lower than the Cellina di Nardò, a cultivar known for its good adaptability to drought environments. In *Xf*-positive plants, RWC value decreased in both cultivars but more significantly in the *Xf*-susceptible Cellina di Nardò, where it was about 46% lower than Ctrl, indicating a severe effect of the pathogen on its water status. The drought/pathogen combined stress further affects *cv* Cellina di Nardò that reached an RWC of about 34%. Conversely, there was no additive effect of water stress and *Xf* on RWC in Leccino (Table 2).

### 3.2. Determination of Carbohydrate Content

**Figure 2:**
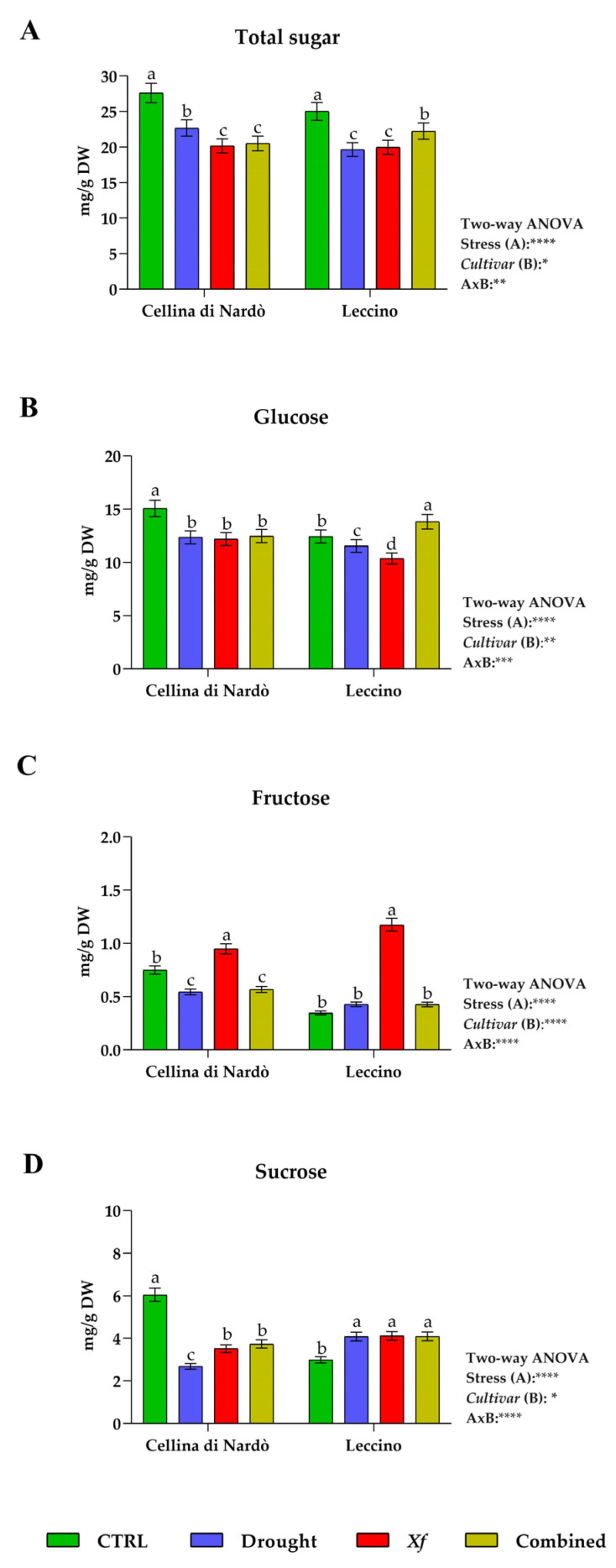
Total sugar (**A**), content of glucose (**B**), fructose (**C**), and sucrose (**D**) expressed as mg/g DW in Cellina di Nardò and Leccino leaves under different conditions: control (Ctrl), water deficit (Drought), *Xylella fastidiosa* (*Xf*), and a combination of water deficit and *Xf* (Combined). Within cvs., different letters correspond to statistically different means carried out using ANOVA followed by the Tukey-HSD post hoc test. Bottom right, two-way ANOVA results were reported.

In the Cellina di Nardò plant, the amount of soluble sugars detected is higher in Ctrl plants in comparison to the other stress conditions (Figure 2A); in fact, the values are lower at 17.9%, 27,0%, 25.7%, respectively, under drought, *Xf*, and combined stress than Ctrl. In Leccino, a decrease in the total sugar amount was also observed in stress conditions compared to Ctrl, but the reduction was settled at about 20% under drought or *Xf*, and about 11% under combined stress.

Glucose, fructose, and sucrose content changes were observed under different conditions (Figure 2B–D). For the Cellina di Nardò, each of the treatments caused a reduction of approximately 18% in glucose compared with the control. Conversely, the combination of drought and *Xf* caused a more significant accumulation of glucose for the Leccino, where an increase of 11.2% compared to Ctrl was observed. Similar to what occurs in the Cellina di Nardò, individual stressors caused a decrease in glucose content. In both cultivars the fructose level is low compared to glucose under all conditions; however, the pathogen causes in both cultivars an increase of fructose: of 26.3% in Cellina di Nardò and more than 200% in Leccino plants in comparison to *Xf*-negative and irrigated plants. Concerning sucrose content, in the Cellina di Nardò, all stresses cause a significant decrease compared to Ctrl, with a higher decrease under water stress conditions, where a reduction of 55.6% was observed. Conversely, the Leccino shows an opposite behavior because all stress conditions caused an increase in sucrose by about 37%.

### 3.3. Expression of the Selected Genes

The expression level of sugar accumulation-related key genes in Cellina di Nardò and Leccino plants was examined, valuating the transcriptional changes of *OeSUT1*, *OeMST2*, *OeSUSY*, *OeGBBS1*, *OeINV-CW*, and *OeINV-V* by RT-qPCR under drought, pathogen, and combined stress.

**Figure 3:**
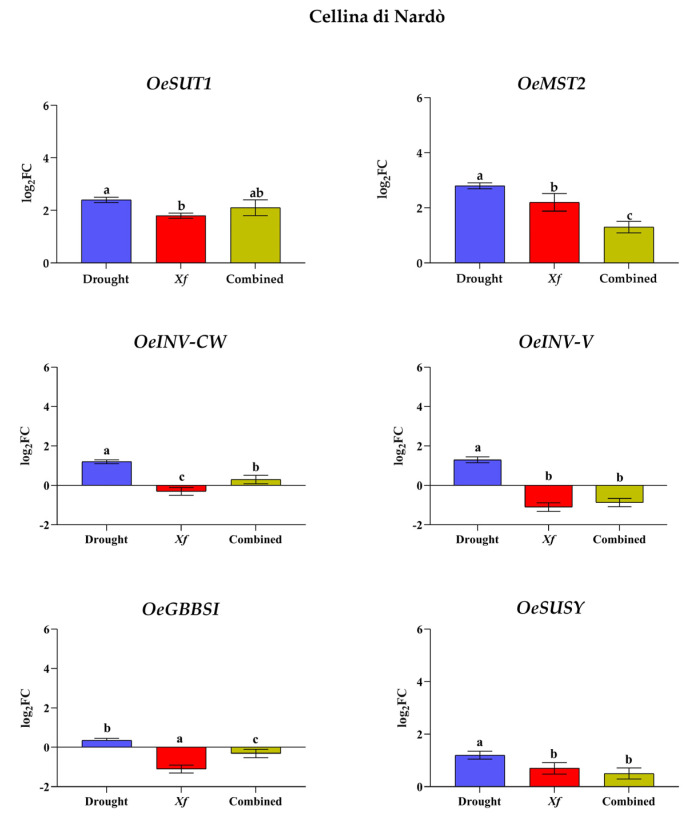
Expression analysis genes involved in sugar metabolism and transport in leaves of Cellina di Nardò cultivar subjected to stresses: drought, pathogen *Xylella fastidiosa*, and combination of both, expressed as log_2_ fold change (log_2_FC) relative to controls. Quantitative analyses of expression of genes coding for the sucrose transporter (*OeSUT1*); the monosaccharide transporter (*OeMST2)*; the cell wall invertase (*OeINV-CW*); the vacuolar invertase (*OeINV-V*); the granule-bound starch synthase I (*OeGBSSI*); the sucrose synthase (*OeSUSY*). Statistical analysis was carried out through one-way ANOVA with Tukey-HSD post hoc test. Within cvs., different letters correspond to statistically different means.

In the Cellina di Nardò (Figure 3), drought stress promoted the transcript levels of all analyzed genes, and those involved in the direct transport of sugars (*OeSUT1* and *OeMST2*) showed the higher up-regulation with log_2_ FC values of 2.4 and 2.8, respectively. These transport-related genes are also up-regulated in *Xf*-positive plants or combined stress, but lower log_2_ FC values were observed compared to drought. Conversely, starch synthase (*OeGBBSI*) and invertase (*OeINV-CW*, *OeINV-V*) were down-regulated in case of *Xf* infection. The drought stress in combination with the presence of the pathogen caused a less significant down-regulation of the starch synthase gene with a log_2_ FC value of −0.3 instead of ≈−1 for *Xf*-positive plants. An opposite behavior was also observed for the *OeINV-CW* gene, but the variation in expression is minimal for both stress conditions compared to Ctrl.

**Figure 4:**
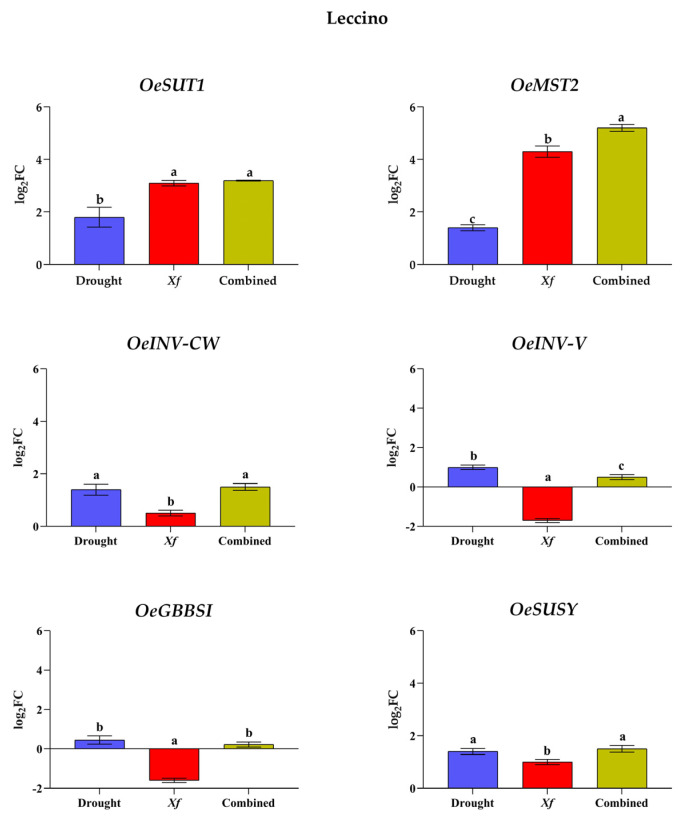
Expression analysis genes involved in sugar metabolism and transport in leaves of Leccino cultivar subjected to stresses: drought, pathogen *Xylella fastidiosa*, and a combination of both, expressed as log_2_ fold change (log_2_FC) relative to controls. Quantitative analyses of expression of genes coding for the sucrose transporter (*OeSUT1*); the monosaccharide transporter (*OeMST2)*; the cell wall invertase (*OeINV-CW*); the vacuolar invertase (*OeINV-V*); the granule-bound starch synthase I (*OeGBSSI*); the sucrose synthase (*OeSUSY*). Statistical analysis was carried out through one-way ANOVA with Tukey-HSD post hoc test. Within cvs., different letters correspond to statistically different means.

In Leccino cultivar, the genes analyzed are triggered by single or combined stresses (Figure 4). The drought stress causes an up-regulation on all selected genes. Furthermore, the pathogen causes higher expression in both *OeSUT1* and *OeMST2* transporter genes (log_2_ FC values: 3.1 and 4.3, respectively) compared to drought. The addition of drought stress (Combined stresses) did not change the expression of the *OeSUT1* gene in comparison to the *Xf* stress, while the expression level of *OeMST2* increased significantly (log_2_ FC value: 5.2). Similar to that observed for Cellina di Nardò, *Xf* caused a reduction of expression of invertase and starch synthase genes, with greater intensity in down-regulation of *OeINV-V* and *OeGBBSI* (log_2_ FC value: −1.7 and −1.6, respectively). Finally, in the Leccino plant, analysis showed the drought and *Xf* stress combination increases the expression of all the genes involved in sugar metabolism and transport (Figure 4) in comparison to only *Xf* stress.

## 4. Discussion

Many studies have shown the effects of drought stress on plants and that the combination drought-pathogen is one of the most critical stress combinations affecting crop yields worldwide. In Salento area, *Xf* has manifested its destructive effects greatly damaging the olive sector and related activities, causing significant economic losses. It has also significantly modified the landscape and the strong social and cultural ties that the olive tree has on the local population [33]. *Xf* is a bacterium that clogs xylem vessels, leading the plant to severe water stress and later death. There are currently no cures, and among the containment actions, identifying and characterizing resistance traits certainly seems to be the most promising strategy that cannot be separated from an understanding of how plants respond to stress. Recent studies suggest that metabolic pathways are the converging points of plant responses to abiotic and biotic stress interactions [34].

In the Cellina di Nardò cultivar, the stressor factors have significantly impacted the distribution of carbohydrates. In particular, *Xf* and its combination with drought significantly decrease total sugars compared to Ctrl plants. Moreover, Leccino showed lower sugar accumulation in Ctrl plants than *Xf*-susceptible plants but offered a less pronounced reduction of sugar content in stress conditions. The decrease in total sugars in plant tissues following pathogen invasion was also observed in the leaf tissue of tomato plants (*Solanum lycopersicum* L.) infected by *Botrytis cinerea* [35] and in *Arabidopsis* after *Sclerotinia sclerotiorum* infection [36]. However, other studies conducted on pea [37], wheat [38], *Nicotiana tabacum* [39], and *Arabidopsis* [40] have instead highlighted that when a pathogen attacks the plant, there is an increase in sugar levels. These conflicting results suggest that the plant’s response to the infectious attack, in terms of the amount of sugars in the tissues, cannot be generalized and that the response could depend on both the plant and the pathogen. Recently, several research groups have pointed out that to obtain more precise indications on biotic stress tolerance, it is more beneficial to evaluate the relative content of glucose, fructose, and sucrose in order to emphasize connections with diseases [41].

In both analyzed cultivars, the most abundant sugar was glucose. In the Cellina di Nardò, the amount of glucose was higher in Ctrl, while as a result of the different stresses, the amount of glucose decreased on average by 18%. Probably, as reported in the literature, stress conditions cause a malfunction or even the partial destruction of the photosystems, or the repression of some genes involved in photosynthesis [42,43,44,45,46]. Moreover, the decrease of glucose induced by *Xf* can be attributed to the use of sugar as an energy source by the pathogen itself [3]. On the other hand, a different behavior was observed in the Leccino after combined stress due to a more significant accumulation of glucose compared to its level in the other conditions. Thus, the increase in glucose level seems to affect the combined stress response in Leccino plants directly. These results should indicate a better metabolic reprogramming of the Leccino to increase the availability of sugars in the cells and, therefore, favor osmotic adjustment in response to combined stress.

The low accumulation of fructose, irrespective of cultivars and stress condition, seems consistent with the literature because fructose is not related to osmoprotection but appears to be related to the synthesis of secondary metabolites [47]. Although, to date, no fructose-specific signaling pathway has been reported [48]; nevertheless, Zhong et al. [49] have indicated that fructose represses the primary root growth of *Arabidopsis* at low concentrations.

Sucrose is an essential osmoprotectant and energy source of plant cells under drought [50] and pathogen stress [51]. The Cellina di Nardò accumulated more of it in *Xf*-negative and irrigated plants, while the unfavorable conditions caused minor accumulation. In the *Xf*-resistant cultivar Leccino, we observed an opposite behavior, because a more significant accumulation is detected in stressed plants than in the Ctrl plants. The results confirmed that the sugar changes do not follow a static model and vary with the genotype and the stress factor [52]. In addition, it is known that not all soluble sugars play similar metabolic roles in plant under stress conditions.

Various studies have shown that the higher accumulation of sucrose is associated with improved plant tolerance to abiotic stress [53], even if there have been contradictory reports regarding the effect of stress on sugar accumulation. For example, some studies report that sugar content increases [54], while others report it decreases [55], while rises or falls in sugar content are sometimes related to the type of stress [5].

During photosynthesis, sucrose is produced in source leaves and transported throughout the plant by Sucrose Transporters (SUTs), which play an essential role in plant growth and signal transduction in the stress response [56,57]. The *OeSUT1* gene is induced by water stress in both olive cultivars, and usually, increased expression of the *SUTs* genes is linked to the ability of the plant to activate when subjected to a water deficit. An increase in the accumulation of transcripts has also been observed in *Arabidopsis* [58] and apple trees (*Malus domestica* Borkh) with a consequent accumulation of sucrose in the leaf tissues and a better resistance to water deficiency [59]. Furthermore, the pathogen alone or in combination with water stress induces gene expression in both olive cultivars, but in the case of *Xf*-resistant Leccino, the presence of the bacterium results in even greater regulation of the transporter gene expression. A study conducted on tomatoes showed that the *SlSUT1* gene is overexpressed in case of infection by the fungus *Glomus mosseae* [60] and in *Arabidopsis* after an attack *Meloidogyne incognita* [61], suggesting that this gene may be involved in the biotic stress response.

Regarding the other transporter, the *OeMST2* gene is also induced by all the stresses analyzed in the Cellina di Nardò, but the drought determines a more significant accumulation of transcripts. A study on transgenic *Arabidopsis* showed overexpression of *OsMST* genes in water stress with consequent accumulation of sugars and better resistance to drought than wild-type plants [62]. Hence, the results confirm the ability of the Cellina di Nardò cultivar to reprogram its cellular activities to tolerate drought stress, also confirmed by the RWC value measured. Based on these discussions, we herein hypothesize that MST2 plays a crucial adaptive role in the supply of carbohydrates to fight abiotic stress. Additionally, in the Leccino, the *OeMST2* gene was induced by all the stress conditions, but the expression level was higher in *Xf*-positive plants, with a further additional effect in response to combined stress. Although the roles of monosaccharide transporters in plant defense response are still unclear, it has been hypothesized that monosaccharide transporters are hired to provide energy to fight invasion by microorganisms or, in some cases, to supply energy to pathogens [28]. Probably, the further increase in the expression *OeMST2* gene in Leccino plants subjected to combined stress could be due to better competence to raise the concentration of soluble sugars in the cells and favor osmotic adjustment. In connection to this, it will be interesting to next evaluate the *SWEET* (Sugars Will Eventually be Exported Transporters) genes because some studies have shown that the induction of plant *SWEET* genes by pathogens is correlated with the capacity of pathogens to obtain host-derived sugars for their sustenance [63].

Regarding synthase genes, the *OeSUSY* gene was induced by water stress in both cultivars with a similar level of expression. A connection between the increased activity of this gene and water stress tolerance has been observed in other species such as poplar [64], apple trees [65], and soybeans [66]. On the contrary, a decrease of the activity of *OsSUSY* was observed in rice (*O. sativa* L.) in drought conditions. This suggests that the gene’s activity is not unique but depends on plant organ, severity, and persistence of stress [67]. Moreover, *Xf* and combined stress induce the expression of *OeSUSY*. In studies on grapevines infected with different phytoplasmas, the overexpression of the *VvSUSY* genes was observed in leaves, suggesting that at the infection site there is a greater demand for simple sugars as a consequence of increased energy consumption for the presence of the pathogen, and for the synthesis of metabolites helpful in dealing with the infection [68,69]. On the contrary, down-regulation of the *TaSUSY* gene was observed in wheat plants infected by *Pyricularia oryzae* together with a reduction of photosynthetic performance, suggesting that the increased activity of this gene is necessary to counteract the stress induced by the pathogen [70]. However, it is interesting to note that in the *Xf* resistant Leccino plant, the expression of *OeSUSY* in combined stress is equal or higher than those observed for the other stress conditions.

In the Cellina di Nardò plant, the *OeGBSSI* gene was induced in response to water deficit, but with a very low expression level, and a similar picture was observed in the Leccino. The decreased starch synthesis has been correlated in various studies with improved drought tolerance [71]. It has been observed that the broad bean cultivars (*Phaseolus vulgaris* L.), resistant to water stress degrade more starch than the susceptible cultivars [72]. Furthermore, a lower starch synthesis following water stress was also reported on rice [73], wheat [68,69], *Polytrichum formosum moss* [74], and tree species such as the lychee [75], in which a reduction in *GBSSI* gene expression was detected.

In response to *Xf*, *OeGBSSI* gene was down-regulated in the Cellina di Nardò, suggesting a probable block of the conversion of sucrose to starch, while combined stress caused expression level close to Ctrl. This behavior was confirmed in the Leccino, where the gene was strongly down-regulated in the presence of *Xf*, while the combination *Xf*/drought induced a very slight gene expression similarly to Ctrl. Generally, after an infection, a decrease of the starch content in the infected region was observed [76], associated with down-regulation genes coding the enzymes responsible for starch synthesis. In fact, the pathogens force plants to reallocate the sugars from which they draw nutrition, sugars that are also necessary for the plant’s defense responses [51].

In plants, invertases are involved in sucrose metabolism, determining the split of sucrose to glucose and fructose. The cell wall invertase (INV-CW) and vacuolar invertase (INV-V), in particular, have a fundamental role in response to abiotic and biotic stress; several studies have reported that the invertase activity contributes to the supply of glucose to mitochondria-associated hexokinase and maintains reactive oxygen species (ROS) homeostasis and photosynthetic efficiency [77,78]. Moreover, sucrose cleavage by INV-CW alters the ratio of sucrose to hexoses in the apoplast and can therefore actively modulate sugar signaling [79].

Under water deficit, the *OeINV-CW* and *OeINV-V* genes are induced both in Cellina di Nardò and Leccino plants with similar gene expression profiles. A correlation between water stress and increase of INVs genes expression, suggesting their role in the plant’s resistance to drought was found by some authors [80,81]. In general, increased expression levels of these genes have been reported to act as a part of the stress response in various plant species [82,83,84].

Our analysis indicated that, in the Cellina di Nardò, genes coding for cell wall invertase and vacuolar invertase are down-regulated or expressed in a Ctrl-like manner in response to pathogen and combined stress. This down-regulation was also observed in *Vicia faba*, *Vitis vinifera*, and *O. europea*, and was related to a decrease in the availability of sucrose in the storage compartment [78,85]. However, in the *Xf* resistant Leccino, the combination of *Xf* and water stress induces both genes. In particular, the *OeINV-CW* shows a high expression level and confirms that Leccino is somehow more trained to withstand combined stress compared to the Cellina di Nardò, as observed for transporter and synthase genes.

In conclusion, due to the peculiar behavior of the considered genes in the Leccino under combined stress, we can assume that the pathways involved in drought stress response induce beneficial effects on pathogen resistance in such cultivar. Several studies have shown the ability of plants subjected to drought stress to acquire disease tolerance [86,87]. Additional studies are required to clarify this point, as water deficit usually negatively influences plant physiology; however, this is not always true when plants face a pathogen attack.

## 5. Conclusions

The data obtained in this study have assigned sugars to more of a mediator role between the perception of stress and the expression of resistance genes than a direct effect; it is well known that sugars also act as signaling molecules, acting as primary messengers regarding detection and signal transduction [88,89,90]. The soluble sugars not only play the role of donors of carbon skeletons and respiratory substrates, but they may also induce metabolic signals influencing the expression of many genes [3,91,92,93,94,95]. As reported by Tarkowski et al. [96], soluble sugar signaling and dynamics are crucial for the control of plant development and organogenesis and are also extremely important for coping with biotic and abiotic stresses.

In this study, a differential expression pattern was observed in Leccino, being resistant to *Xf*, as opposed to the Cellina di Nardò cultivar being susceptible to *Xf*, especially in response to combined stress, confirming what was observed in previous work [21]. As reported in the literature, in some cases, plants can prove resistant or tolerant to biotic stresses if they are first acclimatized even moderately with abiotic stress [97]. In the current context of global climate change, this study supports the importance of investigating multiple stress factors, biotic and abiotic, to explore complex drought–disease interaction and identify the key genes that could lead to the development of new strategies to alleviate the effects of stress conditions. It is evident that *Xf*-diseases are closely related to the signaling pathways triggered by the lack of water and biotic stress and how they interact with each other to influence the expression of the genes involved in the plant defense.

## Figures and Tables

**Table 2:** Relative water content (RWC, %) values of Cellina di Nardò and Leccino olive trees under different conditions: control (Ctrl), water deficit (Drought), *Xylella fastidiosa* (*Xf*), and a combination of water deficit and *Xf* (Combined). Different letters correspond to statistically different means (upper case between rows, lower case between columns) carried out using ANOVA followed by the Tukey-HSD post hoc test.

*Cv*	Ctrl	Drought	*Xf*	Combined
**Cellina di Nardò**	97.29% A,a	87.35% A,b	52.45% B,c	33.72% B,d
**Leccino**	97.72% A,a	68.19% B,c	70.97% A,b	66.85% A,d

## Data Availability

All results are included within the article.

## References

[1] Granot D., David-Schwartz R., Kelly G. (2013). Hexose kinases and their role in sugar-sensing and plant development. Front. Plant Sci..

[2] Gangola M.R., Bharathi R., Ramadoss B.R., Wani S.H. (2018). Sugars Play a Critical Role in Abiotic Stress Tolerance in Plants in: Biochemical, Physiological and Molecular Avenues for Combating Abiotic Stress Tolerance in Plants.

[3] Morkunas I., Ratajczak L. (2014). The role of sugar signaling in plant defense responses against fungal pathogens. Acta Physiol. Plant..

[4] Lawas L.M.F., Zuther E., Jagadish S.K., Hincha D.K. (2018). Molecular mechanisms of combined heat and drought stress resilience in cereals. Curr. Opin. Plant Biol..

[5] Dong S., Beckles D.M. (2019). Dynamic changes in the starch-sugar interconversion within plant source and sink tissues promote a better abiotic stress response. J. Plant Physiol..

[6] Pucciariello C., Perata P. (2021). The Oxidative Paradox in Low Oxygen Stress in Plants. Antioxidants.

[7] Pommerrenig B., Ludewig F., Cvetkovic J., Trentmann O., Klemens P.A.W., Neuhaus H.E. (2018). In Concert: Orchestrated Changes in Carbohydrate Homeostasis Are Critical for Plant Abiotic Stress Tolerance. Plant Cell Physiol..

[8] Yamada K., Osakabe Y. (2018). Sugar compartmentation as an environmental stress adaptation strategy in plants. Semin. Cell Dev. Biol..

[9] Saponari M., Boscia D., Nigro F., Martelli G. (2013). Identification of DNA sequences related to *Xylella fastidiosa* in oleander, almond and olive trees exhibiting leaf scorch symptoms in Apulia (Southern Italy). J. Plant Pathol..

[10] Cardinale M., Luvisi A., Meyer J.B., Sabella E., De Bellis L., Cruz A.C., Ampatzidis Y., Cherubini P. (2018). Specific fluorescence in situ hybridization (FISH) test to highlight colonization of xylem vessels by *Xylella fastidiosa* in naturally infected olive trees (*Olea europaea* L.). Front. Plant Sci..

[11] Giampetruzzi A., Morelli M., Saponari M., Loconsole G., Chiumenti M., Boscia D., Savino V.N., Martelli G.P., Saldarelli P. (2016). Transcriptome profiling of two olive cultivars in response to infection by the CoDiRO strain of *Xylella fastidiosa* subsp. pauca. BMC Genom..

[12] Martelli G.P. (2016). The current status of the quick decline syndrome of olive in southern Italy. Phytoparasitica.

[13] Luvisi A., Aprile A., Sabella E., Vergine M., Nicolì F., Nutricati E., Miceli A., Negro C., De Bellis L. (2017). *Xylella fastidiosa* subsp. *pauca* (CoDiRO strain) infection in four olive (*Olea europaea* L.) cultivars: Profile of phenolic compounds in leaves and progression of leaf scorch symptoms. Phytopathol. Mediterr..

[14] Jlilat A., Ragone R., Gualano S., Santoro F., Gallo V., Varvaro L., Mastrorilli P., Saponari M., Nigro F., D’Onghia A.M. (2021). A non-targeted metabolomics study on *Xylella fastidiosa* infected olive plants grown under controlled conditions. Sci. Rep..

[15] Pavan S., Vergine M., Nicolì F., Sabella E., Aprile A., Negro C., Fanelli V., Savoia M.A., Montilon V., Susca L. (2021). Screening of Olive Biodiversity Defines Genotypes Potentially Resistant to *Xylella fastidiosa*. Front. Plant Sci.

[16] Schneider K., van der Werf W., Cendoya M., Maurits M., Navas-Cortes J.A. (2020). Impact of *Xylella fastidiosa* subspecies *pauca* in European olives. Proc. Natl. Acad. Sci. USA.

[17] Morelli M., García-Madero J.M., Jos Á., Saldarelli P., Dongiovanni C., Kovacova M., Saponari M., Baños Arjona A., Hackl E., Webb S. (2021). *Xylella fastidiosa* in Olive: A Review of Control Attempts and Current Management. Microorganisms.

[18] Alfio M.R., Balacco G., Parisi A., Totaro V., Fidelibus M.D. (2020). Drought index as indicator of salinization of the Salento aquifer (Southern Italy). Water.

[19] Marra F.P., Marino G., Marchese A., Caruso T. (2016). Effects of different irrigation regimes on a super-high-density olive grove cv. “*Arbequina*”: Vegetative growth, productivity and polyphenol content of the oil. Irrig. Sci..

[20] Harper S.J., Ward L.I., Clover G.R.G. (2010). Development of LAMP and Real-time PCR Methods for the rapid detection of *Xylella fastidiosa* for quarantine and field applications. Phytopatology.

[21] De Pascali M., Vergine M., Sabella E., Aprile A., Nutricati E., Nicolì F., Buja I., Negro C., Miceli A., Rampino P. (2019). Molecular Effects of *Xylella fastidiosa* and Drought Combined Stress in Olive Trees. Plants.

[22] Barrs H.D., Weatherley P.E. (1962). A re-examination of the relative turgidity technique for estimanting water deficits in leaves. Aust. J. Biol. Sci..

[23] (2019). PM 7/24 (4) *Xylella fastidiosa*. EPPO Bull..

[24] Chow P.S., Landhausser S.M. (2004). A method for routine of total sugar and starch content in woody plants tissue. Tree Physiol..

[25] Stein O., Granot D. (2019). An overview of sucrose synthases in plants. Front. Plant Sci..

[26] Koch K. (1996). Carbohydrate-modulated gene expression in plants. Annu. Rev. Plant Physiol. Plant Mol. Biol..

[27] Zeeman S.C., Kossmann J., Smith A.M. (2010). Starch: Its metabolism, evolution, and biotechnological modification in plants. Annu. Rev. Plant Biol..

[28] Slewinski T.L. (2011). Diverse functional roles of monosaccharide transporters and their homologs in vascular plants: A physiological perspective. Mol. Plant..

[29] Livak K.J., Schmittgen T.D. (2011). Analysis of relative gene expression data using real-time quantitative PCR and the 2^−∆∆CT^ method. Methods.

[30] Rossi L., Borghi M., Francini M., Lin X., Xie D., Sebastiani L. (2016). Salt stress induces differential regulation of the phenylpropanoid pathway in *Olea europaea* cultivars Frantoio (salt-tolerant) and Leccino (salt-sensitive*)*. J. Plant. Physiol..

[31] Sabella E., Aprile A., Genga A., Siciliano T., Nutricati E., Nicolì F., Vergine M., Negr C., De Bellis L., Luvisi A. (2019). Xylem cavitation susceptibility and refilling mechanisms in olive trees infected by *Xylella fastidiosa*. Sci. Rep..

[32] Alagna F., Cirilli M., Galla G., Carbone F., Daddiego L., Facella P., Lopez L., Colao C., Mariotti R., Cultrera N. (2016). Transcript Analysis and Regulative Events during Flower Development in Olive (*Olea europaea* L.). PLoS ONE.

[33] Semeraro T., Gatto E., Buccolieri R., Vergine M., Gao Z., De Bellis L., Luvisi A. (2019). Changes in olive urban forests infected by *Xylella fastidiosa*: Impact on microclimate and social health in urban areas. Int. J. Environ. Res. Public Health.

[34] Isah T. (2019). Stress and defense responses in plant secondary metabolites production. Biol. Res..

[35] Berger S., Papadopoulos M., Schreiber U., Kaiser W., Roitsch T. (2004). Complex regulation of gene expression, photosynthesis and sugar levels by pathogen infection in tomato. Physiol. Plant..

[36] Chen J., Ullah C., Giddings Vassão D., Reichelt M., Gershenzon J., Hammerbacher A. (2021). *Sclerotinia sclerotiorum* infection triggers changes in primary and secondary metabolism in *Arabidopsis thaliana*. Phytopathology.

[37] Aked J., Hall J.L. (1993). Effect of powdery mildew infection on concentrations of apoplastic sugars in pea leaves. New Phytol..

[38] Wright D.P., Baldwin B.C., Shephard M.C., Scholes J.D. (1995). Source-sink relationships in wheat leaves infected with powdery mildew: 1. Alterations in carbohydrate metabolism. Physiol. Mol. Plant Pathol..

[39] Herbers K., Takahata Y., Melzer M., Mock H.P., Hajirezaei M., Sonnewald U. (2000). Regulation of carbohydrate partitioning during the interaction of potato virus Y with tobacco. Mol. Plant Pathol..

[40] Chou H., Bundock N., Rolfe S., Scholes J. (2000). Infection of *Arabidopsis thaliana* leaves with *Albugo candida* causes a reprogramming of host metabolism. Mol. Plant Pathol..

[41] Lecompte F., Abro M.A., Nicot P.C. (2013). Can plant sugars mediate the effect of nitrogen fertilization on lettuce susceptibility to two necrotrophic pathogens: *Botrytis cinerea* and *Sclerotinia sclerotiorum*?. Plant Soil..

[42] Lecompte F., Nicot P.C., Ripoll J., Abro M.A., Raimbault A.K., Lauri F.L., Bertin N. (2017). Reduced susceptibility of tomato stem to the necrotrophic fungus *Botrytis cinerea* is associated with a specific adjustment of fructose content in the host sugar pool. Ann. Bot..

[43] Hui D., Iqbal J., Lehmann K., Gase K., Saluz H.P., Baldwin I.T. (2003). Molecular interactions between the specialist herbivore *Manduca sexta* (lepidoptera, sphingidae) and its natural host *Nicotiana attenuata*: V. microarray analysis and further characterization of large-scale changes in herbivore-induced mRNAs. Plant Physiol..

[44] Zimmerli L., Stein M., Lipka V., Schulze-Lefert P. (2004). Somerville, S. Host and non-host pathogens elicit different jasmonate/ethylene responses in *Arabidopsis*. Plant J..

[45] de Torres Zabala M., Littlejohn G., Jayaraman S., Studholme D., Bailey T., Lawson T., Tillich M., Licht D., Bölter B., Delfino L. (2015). Chloroplasts play a central role in plant defence and are targeted by pathogen effectors. Nat. Plants.

[46] Guidi L., Lo Piccolo E., Landi M. (2019). Chlorophyll Fluorescence, Photoinhibition and Abiotic Stress: Does it Make Any Difference the Fact to Be a C3 or C4 Species?. Front. Plant Sci..

[47] Rosa M., Prado C., Podazza G., Interdonato R., González J.A., Hilal M., Prado F.E. (2009). Soluble sugars metabolism, sensing and abiotic stress: A complex network in the life of plants. Plant Signal Behav..

[48] Li P., Wind J.J., Shi X., Zhang H., Hanson J., Smeekens S.C., Teng S. (2011). Fructose sensitivity is suppressed in *Arabidopsis* by the transcription factor ANAC089 lacking the membrane-bound domain. Proc. Natl. Acad. Sci. USA.

[49] Zhong Y., Xie J., Wen S., Wu W., Tan L., Lei M., Shi H., Zhu J.K. (2020). TPST is involved in fructose regulation of primary root growth in *Arabidopsis thaliana*. Plant Mol. Biol..

[50] Bolouri-Moghaddam M.R., Le Roy K., Xiang L., Rolland F., Van den Ende W. (2010). Sugar signalling and antioxidant network connections in plant cells. FEBS J..

[51] Tauzin A.S., Giardina T. (2014). Sucrose and invertases, a part of the plant defense response to the biotic stresses. Front. Plant Sci..

[52] Morsy M.R., Jouve L., Hausman J.F., Hoffmann L., Stewart J.M. (2007). Alteration of oxidative and carbohydrate metabolism under abiotic stress in two rice (*Oryza sativa* L.) genotypes contrasting in chilling tolerance. J. Plant Physiol..

[53] Du Y., Zhao Q., Chen L., Yao X., Zhang W., Zhang B., Xie F. (2020). Effect of drought stress on sugar metabolism in leaves and roots of soybean seedlings. Plant Physiol. Biochem..

[54] Li C., Liu Y., Tian J., Zhu Y., Fan J. (2020). Changes in sucrose metabolism in maize varieties with different cadmium sensitivities under cadmium stress. PLoS ONE.

[55] Zhao H., Guan J., Liang Q., Zhang X., Hu H., Zhang J. (2021). Effects of cadmium stress on growth and physiological characteristics of *Sassafras* seedlings. Sci. Rep..

[56] Noiraud N., Delrot S., Lemoine R. (2000). The sucrose transporter of celery: Identification and expression during salt stress. Plant Physiol..

[57] Lemoine R., La Camera S., Atanassova R., Dedaldechamp F., Allario T., Pourtau N., Bonnemai J.L., Laloi M., Coutos-Thevenot P., Maurousset L. (2013). Source-to-sink transport of sugar and regulation by environmental factors. Front. Plant Sci..

[58] Jia W., Zhang L., Wu D., Liu S., Gong X., Cui Z., Cui N., Cao H., Rao L., Wang C. (2015). Sucrose transporter AtSUC9 Mediated by a low sucrose level is involved in *Arabidopsis* abiotic stress resistance by regulating sucrose distribution and ABA accumulation. Plant Cell Physiol..

[59] Ma Q.J., Sun M.H., Lu J., Kang H., You C.X., Hao Y.J. (2019). An apple sucrose transporter MdSUT2.2 is a phosphorylation target for protein kinase MdCIPK22 in response to drought. Plant Biotech. J..

[60] Boldt K., Pörs Y., Haupt B., Bitterlich M., Kühn C., Grimm B., Franken P. (2011). Photochemical processes, carbon assimilation and RNA accumulation of sucrose transporter genes in tomato arbuscular mycorrhiza. J. Plant Physiol..

[61] Hofmann J., Youssef-Banora M., De Almeida-Engler J., Grundler F.M. (2010). The role of callose deposition along plasmodesmata in nematode feeding sites. Mol. Plant Microbe Interact..

[62] Monfared H., Chew J.K., Azizi P., Xue G., Ee S., Kadkhodaei S., Hedayati P., Ismail I., Zainal Z. (2020). Overexpression of a rice monosaccharide transporter gene (*OsMST6*) confers enhanced tolerance to drought and salinity stress in *Arabidopsis thaliana*. Plant Mol. Biol. Rep..

[63] Breia R., Conde A., Badim H., Fortes A.M., Geros H., Granell A. (2021). Plant SWEETs: From sugar transport to plant-pathogen interaction and more unexpected physiological roles. Plant Physiol..

[64] Pelah D., Wang W., Altman A., Shoseyov O., Bartels D. (1997). Differential accumulation of water stress related proteins, sucrose synthase and soluble sugars in *Populus* species that differ in their water stress response. Physiol. Plant..

[65] Yang J., Zhang J., Li C., Zhang Z., Ma F., Li M. (2019). Response of sugar metabolism in apple leaves subjected to short-term drought stress. Plant Physiol. Biochem..

[66] Du Y., Zhao Q., Chen L., Yao X., Zhang H., Wu J., Xie F. (2020). Effect of drought stress during soybean R2-R6 growth stages on sucrose metabolism in leaf and seed. Inter. J. Mol. Sci..

[67] Xu W., Cui K., Xu A., Nie L., Huang J., Peng S. (2015). Drought stress condition increases root to shoot ratio via alteration of carbohydrate partitioning and enzymatic activity in rice seedlings. Acta Physiol. Plant..

[68] Hren M., Ravnikar M., Brzin J., Ermacora P., Carraro L., Bianco P.A., Casati P., Borgo M., Angelini E., Rotter A. (2009). Induced expression of sucrose synthase and alcohol dehydrogenase I genes in phytoplasma-infected grapevine plants grown in the field. Plant Pathol..

[69] Choi Y.H., Tapias E.C., Kim H.K., Lefeber A.W., Erkelens C., Verhoeven J.T., Brzin J., Zel J., Verpoorte R. (2004). Metabolic discrimination of *Catharanthus roseus* leaves infected by phytoplasma using 1H-NMR spectroscopy and multivariate data analysis. Plant Physiol..

[70] Rios J.A., Rios V.S., Aucique Pérez C.E., Cruz M.F.A., Morais L.E., DaMatta F.M., Rodrigues F.A. (2017). Alteration of photosynthetic performance and source–sink relationships in wheat plants infected by *Pyricularia oryzae*. Plant Pathol..

[71] Thalmann M., Santelia D. (2017). Starch as a determinant of plant fitness under abiotic stress. New Phytol..

[72] González-Cruz J., Pastenes C. (2012). Water-stress-induced thermotolerance of photosynthesis in bean (*Phaseolus vulgaris* L.) plants: The possible involvement of lipid composition and xanthophyll cycle pigments. Environ. Exp. Bot..

[73] Wang Z., Quebedeaux B., Stutte G.W. (1996). Partitioning of (14C) glucose into sorbitol and other carbohydrates in apple under water stress. Aust. J. Plant Physiol..

[74] Yi B., Zhou Y., Gao M., Zhang Z., Yi H., Yang G., Xu W., Huang R. (2014). Effect of drought stress during flowering stage on starch accumulation and starch synthesis enzymes in sorghum grains. J. Integr. Agric..

[75] Lu H., Hu Y., Wang C., Liu W., Ma G., Han Q., Ma D. (2019). Effects of High Temperature and drought stress on the expression of gene encoding enzymes and the activity of key enzymes involved in starch biosynthesis in wheat grains. Front. Plant Sci..

[76] Pressel S., Ligrone R., Duckett J.G. (2006). Effects of de- and rehydration on food conducting cells in the moss *Polytrichum formosum*: A cytological study. Ann. Bot..

[77] Xiang L., Le Roy K., Bolouri-Moghaddam M.R., Vanhaecke M., Lammens W., Rolland F., Van den Ende W. (2011). Exploring the neutral invertase–oxidative stress defense connection in *Arabidopsis thaliana*. J. Exp. Bot..

[78] Dahro B., Wang F., Peng T., Liu J.H. (2016). *PtrA/NINV*, an alkaline/neutral invertase gene of *Poncirus trifoliata*, confers enhanced tolerance to multiple abiotic stresses by modulating ROS levels and maintaining photosynthetic efficiency. BMC Plant Biol..

[79] Proels R.K., Hückelhoven R. (2014). Cell-wall invertases, key enzymes in the modulation of plant metabolism during defence responses. Mol. Plant Pathol..

[80] Villadsen D., Rung J.H., Nielsen T.H. (2005). Osmotic stress changes carbohydrate partitioning and fructose-2,6-bisphosphate metabolism in barley leaves. Funct. Plant Biol..

[81] Storr T., Hall J.L. (1992). The effect of infection by *Erysiphe pisi* DC on acid and alkaline invertase activities and aspects of starch biochemistry in leaves of *Pisum sativum* L. New Phytol..

[82] Pelleschi S., Rocher J.P., Prioul J.L. (1997). Effect of water restriction on carbohydrate metabolism and photosynthesis in mature maize leaves. Plant Cell Environ..

[83] Kim J.Y., Mahé A., Brangeon J., Prioul J.L. (2000). A maize vacuolar invertase, IVR2, is induced by water stress. Organ/tissue specificity and diurnal modulation of expression. Plant Physiol..

[84] Albacete A., Cantero-Navarro E., Großkinsky D.K., Arias C.L., Balibrea M.E., Bru R. (2015). Ectopic overexpression of the cell wall invertase gene CIN1 leads to dehydration avoidance in tomato. J. Exp. Bot..

[85] Koonjul P.K., Minhas J.S., Nunes C., Sheoran I.S., Saini H.S. (2005). Selective transcriptional down-regulation of anther invertases precedes the failure of pollen development in water-stressed wheat. J. Exp. Bot..

[86] Voegele R.T., Wirsel S., Möll U., Lechner M., Mendgen K. (2006). Cloning and characterization of a novel invertase from the obligate biotroph Uromyces fabae and analysis of expression patterns of host and pathogen invertases in the course of infection. Mol. Plant–Microbe Interact..

[87] Hayes M.A., Feechan A., Dry I.B. (2010). Involvement of abscisic acid in the coordinated regulation of a stress-inducible hexose transporter (VvHT5) and a cell wall invertase in grapevine in response to biotrophic fungal infection. Plant Physiol..

[88] Conrath U., Beckers G.J., Flors V., García-Agustín P., Jakab G., Mauch F., Newman M.-A., Pieterse C.M.J., Poinssot B., Pozo M.J. (2006). Priming: Getting ready for battle. Mol. Plant-Microbe Interact..

[89] Barradas C., Pinto G., Correia B., Castro B., Phillips A.J., Alves A. (2018). Drought x disease interaction in *Eucalyptus globulus* under *Neofusicoccum eucalyptorum* infection. Plant Pathol..

[90] Eom J.S., Chen L.Q., Sosso D., Julius B.T., Lin I.W., Qu X.Q., Braun D.M., Frommer W.B. (2015). SWEETs, transporters for intracellular and intercellular sugar translocation. Curr. Opin. Plant Biol..

[91] Yoon J., Cho L.H., Tun W., Jeon J.S., An G. (2021). Sucrose signaling in higher plants. Plant Sci..

[92] Li L., Liu K.H., Sheen J. (2021). Dynamic Nutrient Signaling Networks in Plants. Annu. Rev. Cell Dev. Biol..

[93] Rolland F., Baena-Gonzalez E., Sheen J. (2006). Sugar sensing and signaling in plants: Conserved and novel mechanisms. Annu. Rev. Plant Biol..

[94] Bolouri Moghaddam M.R., Van den Ende W. (2013). Sweet immunity in the plant circadian regulatory network. J. Exp. Bot..

[95] Trouvelot S., Héloir M.C., Poinssot B., Gauthier A., Paris F., Guillier C., Combier M., Trdá L., Daire X., Adrian M. (2014). Carbohydrates in plant immunity and plant protection: Roles and potential application as foliar sprays. Front. Plant Sci..

[96] Tarkowski Ł.P., Van de Poel B., Höfte M., Van den Ende W. (2019). Sweet immunity: Inulin boosts resistance of lettuce (*Lactuca sativa*) against grey mold (*Botrytis cinerea*) in an ethylene-dependent manner. Int. J. Mol. Sci..

[97] Ramegowda V., Senthil-Kumar M., Ishiga Y., Kaundal A., Udayakumar M., Mysore K.S. (2013). Drought stress acclimation imparts tolerance to *Sclerotinia sclerotiorum* and *Pseudomonas syringae* in *Nicotiana benthamiana*. Int. J. Mol. Sci..

